# Influence of Low Dietary Inclusion of the Microalga *Nannochloropsis gaditana* (Lubián 1982) on Performance, Fish Morphology, and Muscle Growth in Juvenile Gilthead Seabream (*Sparus aurata*)

**DOI:** 10.3390/ani10122270

**Published:** 2020-12-01

**Authors:** María Dolores Ayala, Carolina Galián, Victoria Fernández, Elena Chaves-Pozo, Daniel García de la Serrana, María Isabel Sáez, Alba Galafaz Díaz, Francisco Javier Alarcón, Tomás Francisco Martínez, Marta Arizcun

**Affiliations:** 1 Department of Anatomy and Comparative Pathological Anatomy, Faculty of Veterinary, Campus of Espinardo, University of Murcia, 30100 Murcia, Spain; carolina.galian@um.es (C.G.); victoria.fernandez5@um.es (V.F.); 2 Instituto Español de Oceanografía, Centro Oceanográfico de Murcia, Puerto de Mazarrón, 30860 Murcia, Spain; elena.chaves@ieo.es (E.C.-P.); marta.arizcun@ieo.es (M.A.); 3 Department of Cell Biology, Physiology and Immunology, Faculty of Biology, University of Barcelona, 08028 Barcelona, Spain; garciadelaserrana@ub.edu; 4 Departamento de Biología y Geología, Universidad de Almería, CEIMAR, 04120 Almería, Spain; msc880@ual.es (M.I.S.); albagalafat@gmail.com (A.G.D.); falarcon@ual.es (F.J.A.); tomas@ual.es (T.F.M.)

**Keywords:** growth, microalgae fed groups, muscle cellularity, *Sparus aurata*

## Abstract

**Simple Summary:**

Currently plant products are used to partially replace fish meals and oils in fish diets. However, the excessive use of these products can cause nutritional imbalances and environmental problems in their production. For this reason, microalgae appear as an alternative, since they have a high nutritional value and improve the immune status of fish. In the present work *Nannochloropsis gaditana* was included at 2.5% and 5% in substitution of plant products to observe its influence on the growth and morphology of gilthead seabream at low inclusion levels. Furthermore, cellulases were used to degrade cell walls and to increase the bioavailability of the intracellular bioactive compounds. The results showed that the inclusion of *N. gaditana* at low levels in the raw state was sufficient to obtain optimum growth, so it can be used as a partial substitute of vegetables in gilthead seabream diets, without substantially increasing the cost of the feed.

**Abstract:**

A 90-d feeding trial was conducted in which five groups of gilthead seabream (11.96 g initial body weight) were fed with a microalgae-free diet (control group, C) or four diets containing the microalgae *Nannochloropsis gaditana* at two inclusion levels (2.5% or 5%), either raw (R2.5 and R5 batches) or cellulose-hydrolyzed (H2.5 and H5 batches), to study their effect on the body and muscle growth. At 40 days, the highest values of body length and weight were reached in R5 group, but at 64 and 90 days, these were reached in R2.5. However, feed conversion rate, specific growth, daily intake, and survival (100%) were similar in all the groups. The acquisition of a discoid body shape was accelerated depending on the inclusion level of *N. gaditana* in the diets. Moreover, H5 diet affected the fish geometric morphology compared to R5 diet. The white muscle transverse area was similar in all groups at 40 days, with the exception of H2.5 group, which showed the lowest area. At day 90, C and R2.5 displayed the highest muscle growth, attributable to increased hyperplasia in C, and higher hypertrophy in R2.5. However, the highest proportion of small and medium fibers was observed in R5 and H5.

## 1. Introduction

The use of plant proteins has allowed the aquaculture industry to grow without increasing the pressure on wild fisheries. However, the sustainability of land-based protein sources is debatable as these compete with land area for human food production and depend on the use of fresh water [1]. In addition, complete replacement of fishmeal with plant proteins has proven difficult for many species, particularly for marine fish [2,3]. Compared to fishmeal and fish oil, terrestrial plant ingredients show imbalanced amino acid composition, high levels of carbohydrates, and potential presence of anti-nutritional compounds that may lead adverse effects on gut health, digestion, and utilization of nutrients [4,5,6]. Furthermore, feeds based exclusively on plant ingredients can produce a deficit in the dietary contribution of eicosapentaenoic acid (EPA) and docosahexaenoic acid (DHA) to fish.

Soybean derivatives are regularly used as feedstuffs in aquafeed manufacturing for partial replacement of fishmeal and fish oil [2,3]. Dehulled solvent-extracted soybean meal is a high-quality protein source with steady supply and competitive costs. However, the soybean farming areas have become a major driver for worldwide deforestation and loss of biodiversity in developing countries, along with other environmental and social concerns [7].

In this context, microalgae have emerged as a promising resource in the last years. Microalgae have high protein and polyunsaturated fatty acid content, as well as pigments and vitamins that confer high nutritional value for fish. In addition, microalgae improve the immunity status of fish and are beneficial for their growth and health [8,9]. A considerable research effort is being carried out on this subject, which has shown that, for instance, *Arthrospira* sp., *Tetraselmis* sp., or *Isochrysis* sp. could partially replace fish meal in diets for tilapia (*Oreochromis mossambicus*), Atlantic salmon (*Salmo salar*), common carp (*Cyprinus carpio*), and sea bass (*Dicentrarchus labrax*) [10,11,12,13]. Other microalgae species were able to successfully replace fishmeal in the range of 6 to 20%, for example, *Scenedesmus almeriensis*, *Tetraselmis suecica*, and *Tisochrysis lutea* in gilthead seabream (*Sparus aurata*) [14,15]. 

In the present work, we studied the influence of diets with low inclusion level of *Nannochloropsis gaditana* on the biometric parameters and muscle growth of gilthead seabream (*Sparus aurata*) juveniles throughout a 90-d feeding trial. *N. gaditana* is a microalgae belonging to the *Eustigmatophyceae* class, whose species are characterized by their high content of poly- unsaturated fatty acids (PUFAs), especially EPA, arachidonic acid (ARA), and DHA, of great importance in the nutrition of marine animals, especially in the growth and development of fish larvae [16,17]. *N. gaditana* is found mainly in the marine environment, but it can also be found in fresh and brackish waters [18]. Different species of *Nannochloropsis* have shown positive results in teleost fish when are included in aquafeeds. Thus, defatted *N. oceanica* has been tested on post-smolt Atlantic salmon, at a modest 10% inclusion level, with no adverse effects on fish health [19]. The dietary inclusion of 30% *N. oceanica* in post-smolt Atlantic salmon diets, has also shown promising results in terms of feed digestibility [20]. Similarly, in Nile tilapia (*Oreochromis niloticus*), some authors [21] reported higher protein retention, as well as improved fatty acid profile, as a result of the inclusion of 50–100% *N. salina* in feeds to replace fishmeal, fish oil, and soy products. 

On the other hand, the bioavailability of microalgae is not always correlated to the level of inclusion in the diet. The latter might be related to the fact that some species of microalgae, such as *Chlorella* sp. and *Nannochloropsis* sp., have a thick cell wall rich in cellulose, which hinders the digestion and absorption of their nutrients. For this reason, in this work, we explored the effects of low inclusion levels of *N. gaditana* (2.5% or 5%), added either raw or hydrolyzed with cellulases. Cellulases were used with the aim of partially to degrade cell walls and, thereby, to increase the bioavailability of the intracellular bioactive compounds. 

Finally, studies of the influence of microalgae on the muscle growth of fish are still very scarce. The growth of the skeletal muscle involves the recruitment of stem cells and subsequent hypertrophy of muscle fibers [22]. The relative contribution of muscle fibers hypertrophy and hyperplasia to the total muscle growth varies according to endogenous and exogenous factors. One of the most important external factors is the diet [23,24]. One recent study [1] reported that *N. oceanica*-supplemented diets did not influence the fast muscle cellularity of spotted wolffish (*Anarhichas minor*). In the present work, we also studied the influence of *N. gaditana* either raw or hydrolyzed on the muscle cellularity of gilthead seabream, in order to determine its possible influence on this specific aspect, even at low inclusion levels.

## 2. Material and Methods

### 2.1. Animals and Management

This research was carried out on healthy juvenile specimens of gilthead seabream (*Sparus aurata*) obtained in February 2019 from a broodstock breed at the Instituto Español de Oceanografía (Centro Oceanográfico de Murcia, Mazarrón, Spain). All the specimens were kept under the same conditions from hatching to the beginning of the feeding trial. Animals (*n* = 800; 11.96 ± 0.04 g average body weight; 9.64 ± 0.01 cm average body length) were randomly distributed in 5 experimental groups (160 fish group^−1^) consisting of duplicate tanks (2000 L tank^−1^; 80 fish tank^−1^). Fish distributed in the different tanks were homogeneous at the beginning of the experiment (*p* > 0.05). Initial stock density was 0.48 kg m^−3^, and sea water renewal rate (37‰ salinity) was kept at 1000 L h^−1^ in an open flow circuit, maintaining values of ammonia and nitrites (<0.1 mg/L) suitable for gilthead sea bream culture Animals were kept under natural photoperiod and temperature; thus, the water temperature gradually declined from 24 °C at the beginning of the feeding trial (September) to 17 °C during the assay, while the photoperiod initially was 12:12 L:D and progressively changed to 11:13 L:D. The light intensity ranged from 40–60 lux. The tanks were equipped with aerators to maintain an adequate level of oxygenation (above 6 mg/L).

All specimens studied were handled in accordance with the Guidelines of the European Union Council (2010/63/EU), the Committee on the Ethics of Animal Experiments of the IEO (REGA: ES300261040017), and the approval of the Ministry of Water, Agriculture and Environment of the Autonomous Community Region of Murcia (Spain; A13200101).

### 2.2. Experimental Diets

The five experimental diets (Table 1) were manufactured at the Servicio de Dietas Experimentales of the Universidad de Almería (http://www.ual.es/stecnicos_spe). A microalgae-free diet (control, C) plus four diets containing the microalgae *Nannochloropsis gaditana* were elaborated. Two inclusion levels were tested (2.5 or 5% *w/w*), either with the raw microalgae (R2.5 and R5 batches) or with the cellulose-hydrolyzed biomass (H2.5 and H5 groups). All the experimental diets were isoproteic and isolipidic (Table 1). The moisture content ranged from 8 to 9%. The size of the pellets was 2 mm in all diets.

*N. gaditana* was cultured in tubular photobioreactors at the pilot plant (EU-H2020 SABANA facilities funded by the grant # 727874) of the Universidad de Almería (Spain). The chemical composition of *N. gaditana* was 44.5% crude protein, 33.3% carbohydrates, 4.5% ash, and 17.7% crude lipids, which include 35.8% EPA. In order to prepare the diets containing the hydrolyzed microalgae (H2.5 and H5 diets), hydrolysis of algae was produced starting from a sludge containing up to 150 g L^−1^ of biomass after performing an enzymatic hydrolysis with a commercial cellulase (22178, Sigma-Aldrich, Madrid, Spain) under controlled conditions (pH 5.0 and 50 °C under continuous stirring) for 4 h, providing 2% (*w/w*) cellulose. The hydrolysis of *N. gaditana* under these conditions was confirmed by assessing the amount of free amino acids [25], reducing sugars [26], and protein [27] released to the reaction vessel, as well as by microscopic observation of the microalgae after the procedure. Following hydrolysis, the mixture was heated at 80 °C for 15 min for inactivating the cellulolytic enzymes, and then used for manufacturing aquafeeds.

All the animals were fed ad libitum three times a day with a maximum intake of 3% their biomass day^−1^ for 90 days. The amount of diet ingested was recorded daily in each tank.

### 2.3. Sampling

The sampling points were carried out in the following days of the experiment: day 0 (specimens of 11.96 g and 9.64 cm; 6.8 months of age) before being randomly classified in the different experimental groups, day 40 (8.1 months of age), day 64 (8.9 months of age), and day 90 (9.8 months of age), at the end of the experimental period All fish from each tank were collected and sedated with 40 ppm clove oil, and their body length (BL) and weight (BW) were recorded at each sampling point. Then, a total of 60 fish per experimental group (*n* = 30 fish tank^−1^) were photographed with a digital camera (IXUS 700, Canon Madrid, Spain) mounted on a tripod with a light source for morphometric analysis (see below). Finally, 10–12 fish per group were slaughtered by overdose of anesthesia (60 ppm of clove oil), followed by spine severing at days 0, 40, and 90 of the feeding trial, and muscle samples were withdrawn for analyzing muscular growth parameters at the Veterinary Faculty of the University of Murcia as detailed below.

The condition factor (CF) was calculated following Fulton’s K-index (g cm^−3^) according to the following formula: 100 × (body weight/lenght^3^) for each fish, in all the sampling points of the experiment (days 0, 40, 64, and 90). At 40, 64, and 90 days, the following rates were also calculated in all groups: feed conversion rate (FCR): (total feed being consumed/weight gain) and specific growth rate (SGR) (% d^−1^): 100 × {(ln final weight – ln initial weight)/days}. At 64 and 90 days the daily intake rate (DIR) (% d^−1^): 100 × {feed given/{(initial weight + final weight)/2}/days)} was also calculated. The survival rate was calculated at the end of the experiment.

### 2.4. Analysis of the Morphological Parameters

**Figure 1:**
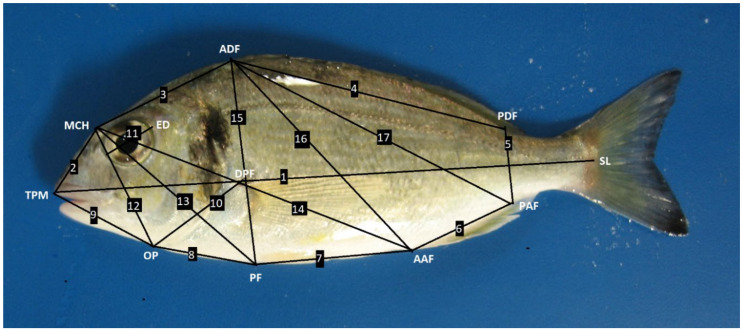
Representation of the seventeen distances measured in fish and used in the morphological analysis described from the subsequent landmarks: the tip of the premaxillar (TPM), the point of the maximum curvature in the head profile curve (MCH), the anterior insertion of the dorsal fin (ADF), the posterior insertion of the dorsal fin (PDF), the anterior insertion of the anal fin (AAF), the posterior insertion of the anal fin (PAF), the anterior insertion of the pelvic fin (PF), the insertion of the operculum in the profile (OP), and the dorsal insertion of the pectoral fin (DPF). The standard length (SL) and the eye diameter (ED) were also included as measure 1 and 11, respectively, in the analysis.

A ruler was included in each fish photograph in order to guarantee a correct calibration in the further image processing carried out by using ImageJ software (National Institutes of Health, Bethesda, MA, USA). A set of 17 different morphological measures was established to generate a box-trust network that enabled a precise definition of fish morphology [28], using 9 different landmarks (Figure 1). The variability in the image calibration among fish was always lower than 0.5%.

### 2.5. Quantitative Analysis of Muscle Growth

After measuring body length (BL) and body weight (BW) of the specimens, these were cut transversely to the long body axis, and then 5-mm thick whole-body slices were obtained. The whole cross muscle sections from each fish were photographed for further morphometric analysis (Sygma-Scan Pro_5 system, Systat Software Inc., San Jose, CA, USA). Subsequently, these body slices were cut into smaller blocks and then snap frozen in 2-methylbutane over liquid nitrogen. Later, sections of 8 μm thickness were obtained from those frozen blocks in a cryostat (Leyca CM 1850, Leica Microsistemas SLU, Barcelona, Spain), and then these sections were stained with hematoxylin-eosin for morphometric studies of the muscle under light microscope. Muscle growth was quantified by means of the morphometric analysis cited above. The total cross-sectional area of the white muscle was measured at 0, 40, and 90 days. In addition, at 0 and 90 days, the following parameters were measured: the number of white muscle fibers (N); the area (A) and minor axis length (D) of white muscle fibers; and muscle fiber density (number of white fibers μm^–2^) (Dens). The average size was estimated from ~ 600 fibers (± 10 sd) located at the intermediate and the apical sectors of the epaxial quadrant of the transversal section of the myotome, according to the methodology described in previous studies [29,30] on this species.

### 2.6. Statistical Analysis

The statistical analysis was performed by means of the statistical package SPSS 24 (IBM, New York, NY, USA). All the data were expressed as mean ± standard error (SEM). Regarding the analysis of the morphology of fish, normality and homogeneity of variances were evaluated using the Kolmogorov–Smirnov and Levene tests, respectively. The non-normal variables were log-transformed. The differences between the mean values were examined using ANOVA and Duncan post-hoc tests or the equivalent nonparametric Kruskal–Wallis test when appropriate. The significance level was 95% in all cases (*p* < 0.05). In order to integrate and to interpret the morphometric measurements performed in the five experimental groups, a principal component analysis (PCA) with varimax rotation was selected because the rotation minimizes the number of variables that have high ladings on a factor. The two principal components (PCs) with higher Eigen-values were considered and interpreted in terms of isometric size variation (PC1) and allometric shape variation (PC2) [31,32,33,34]. ANOVA at *p* < 0.05 was used to test if there were differences between treatments and time.

Regarding the muscle fibers, data distribution was analyzed in each stage by the Shapiro–Wilk test for *p* < 0.05. In relation to the size of the fibers, data did not show a normal distribution (*p* < 0.05) and the Levene’s test did not show homogeneous variances (*p* < 0.05) either. Hence, nonparametric tests (Mann–Whitney and Kolmogorov–Smirnov tests) were used to evaluate the effect of the diet on the size of the fibers, for *p* < 0.05. For most of the other parameters, both tests (Shapiro–Wilk and Levene) showed values of *p* > 0.05; hence, the analysis of variance (ANOVA) were used. However, nonparametric tests were used in the cases with values of *p* < 0.05. Density plots were generated in RStudio [35] using the ggplot2 [36] package.

## 3. Results

### 3.1. Body Growth Parameters and Survival

After 40 days, the highest BL values were reached in the groups R2.5 and R5 (Table 2). However, these differences were not always significant. The highest BW values were reached in R5 group, although significant differences were observed only with regard to H2.5 group. As a consequence of BL and BW values, differences in CF values were also found (Table 2). Thus, R5 group showed the lowest values for CF. FCR and SGR values were similar in all groups.

At 64 days of the feeding trial, the highest BL values were observed in R2.5 and R5 groups (*p* < 0.05), whereas the highest BW values were obtained in R2.5 group (Table 3). The lowest CF values were obtained in R5 batch, but this was only significant in relation to H5. No significant differences were observed in FCR, SGR, and DIR among the different groups (Table 3).

At the end of the experiment (day 90), no significant differences were found for BL and BW values among the experimental groups (*p* > 0.05) (Table 4), although the highest values were obtained in R2.5 group, more noticeably for BW. CF, FCR, SGR, and DIR were similar in all groups (Table 4). Survival was 100% in all dietary treatments.

### 3.2. Fish Morphology Parameters

The seventeen morphometric measurements that were performed in this work produced a box-truss network of linear distances among the morphometric landmarks of gilthead seabream (Figure 1). All the animals, regardless of the dietary treatment, grew up properly during the experimental period, as significant increases in all the measurements were observed over time (Table 5, Table 6 and Table 7 and Figure S1). In addition, most of the measurements also showed significant differences among different treatments, according to a two-way ANOVA test (Figure S1). When data were analyzed with a PCA test, two principal factors, which explained the 99.31% of the total variance, were extracted, and significant differences were attributable to the following variables: treatment, time, and their interaction (Table 8). The first factor (PC1) explained 51.52% of the variance, and the second factor (PC2) explained the 47.78% of the variance, as well as represented the isometric size and the allometric shape, respectively.

**Figure 2:**
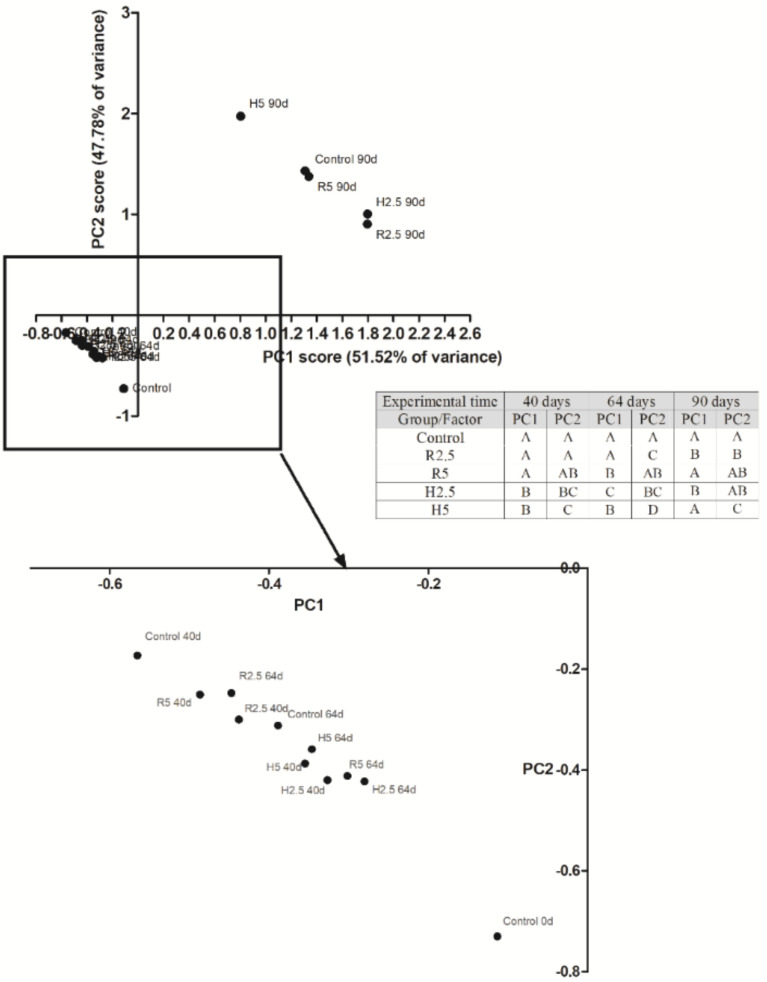
Scatter plot of the mean values of PC1 (isometric size) and PC2 (allometric shape) accordingly with the PCA analysis for the different experimental groups after 0, 40, 64 and 90 days of treatment with a diet containing 2.5 or 5% of raw (R2.5 and R5, respectively) or hydrolyzed (H2.5 or H5, respectively) microalgae biomass or a diet without microalgae biomass (Control). Statistical analysis was performed by two-way ANOVA followed by Duncan’s post-hoc analysis. Inset: Different letters indicate significant differences (*p* < 0.05) among groups.

When data from fish were represented according to these two factors (Figure 2), it could be observed that control fish from day 0 to day 40 decreased their isometric sizes, as they became shorter and wider in shape (PC2 increased), while, at day 64, control fish increased their isometric size, and their shape became larger and narrower. The highest growth was observed in control fish at day 90, according to PC1 and PC2 values (1.31 ± 0.23 and 1.43 ± 0.24, respectively). Fish that had been fed with H5 diet recorded larger isometric size and longer and narrower shape than control fish at days 40 and 64. Fish that had been fed with R5 diet showed larger isometric size at day 64, but such values were similar to those of control group after 90 days. However, the animals that had been fed with H2.5 diet recorded significantly bigger isometric sizes than control fish throughout the complete feeding trial. Interestingly, these fish showed longer and narrower shape at days 40 and 64 compared to control fish, but, after 90 days, no significant differences between H2.5 and control fish were observed (Figure 2). Interestingly, at day 90, the fish that had been fed with R2.5 diet showed bigger isometric sizes and a longer and narrower shapes than control fish. The smallest isometric size, together with the widest and shortest shape, were observed in H5 fish after 90 days (Figure 2).

### 3.3. Muscle Growth

At the beginning of the experiment, all fish randomly distributed in the experimental tanks presented homogeneous values for transverse section of white muscle and muscle cellularity (Table 9 and Table 10).

At day 40, the transverse section of the white muscle of H2.5 group was significantly lower than that of C and R5 groups (*p* < 0.05) (Table 9). After 90 days, the highest value for this parameter was reached in C group (Table 9). Thus, at the end of the experiment (90 days), the values of this parameter showed the following trend: C > R2.5 > R5 > H2.5 > H5. However, differences were significant only between C and H5 batches.

**Figure 3:**
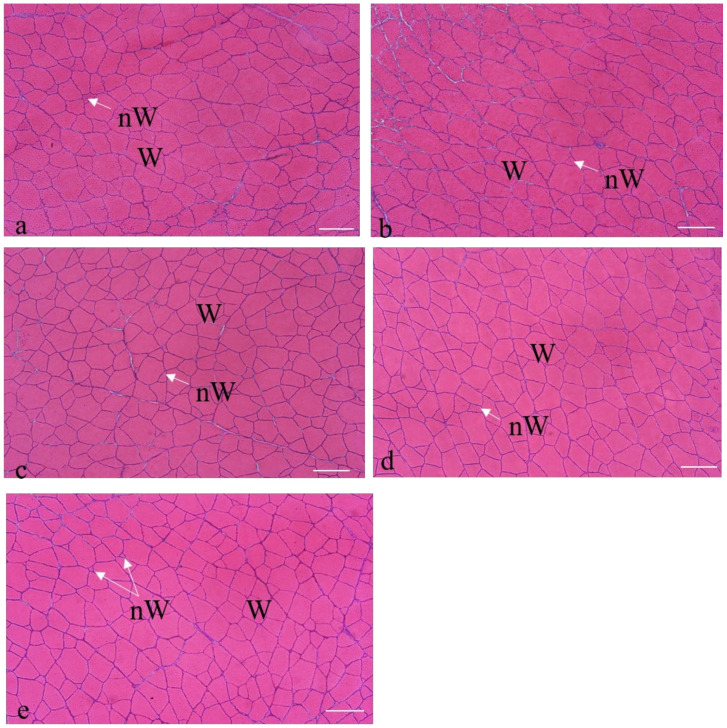
Transverse sections of the white muscle of gilthead seabream specimens fed C (**a**); R2.5 (**b**); R5 (**c**); H2.5 (**d**); and H5 (**e**) diets. Hematoxylin-eosin staining. W: white muscle fibers; nW: new white muscle fibers. Bars 500 µm.

Figure 3 shows the cross section of the white muscle of one specimen from each group, at the end of the feeding trial (90 days). As expected in these stages, the white muscle showed the typical morphological mosaic of post-larval and adult specimens, with small white fibers interposed among big white fibers. C group displayed the highest number of white fibers (hyperplasia), while the lowest hyperplasia was observed in H5 (Figure 3a,e and Table 10). The size of the white fibers (hypertrophy) was bigger in R2.5 fish than in the other fish groups (Figure 3b). The R5 and H2.5 groups showed similar values with each other (Figure 3c,d), with values of the number of white fibers greater than the H5 group but lower than the other groups (Table 10). Their hypertrophy was intermediate between the values that were found in R2.5 and H5. However, the differences between groups in the internal fibrillar constitution of the muscle (hypertrophy and hyperplasia) were not significant (*p* > 0.05) at 90 days (Table 10).

**Figure 4:**
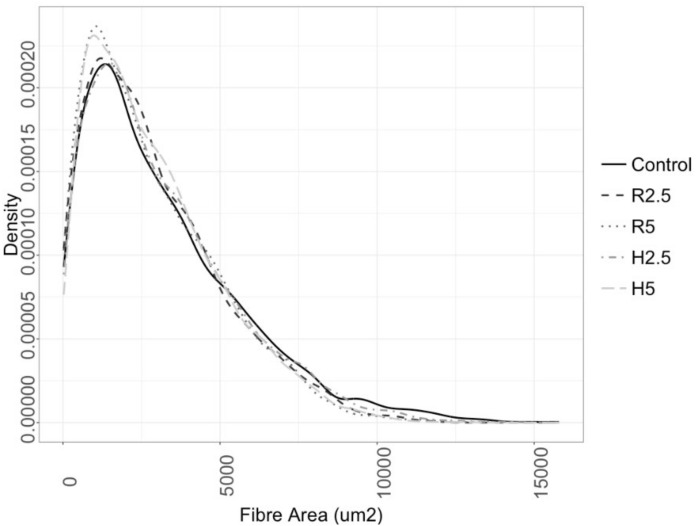
Gilthead seabream fast skeletal muscle fiber area distribution. Fast skeletal fiber area density plots from gilthead seabream fed the experimental diets over 90 days. Dark line represents the average density function of the control group and the different dashed lines represent groups fed R2.5, R5, H2.5, and H5 diets (ordered from black to light grey).

On the other hand, the fibers area distribution showed overlapping profiles between fish of the control group and those with the lowest microalgae inclusion in the diets (R2.5 and H2.5; Figure 4). Fish that had been fed with the highest microalgae inclusion level (R5 and H5) showed higher proportion of fibers size between 200 and 1200 µm^2^ than the other groups (Figure 3), which was parallel to higher values of fibrillar density in these groups than in C and R2.5 (Table 10).

## 4. Discussion

### 4.1. Influence of the Diet on the Body Growth Parameters, Fish Morphology, and Survival

Microalgae are currently used as an alternative protein source to fishmeal, with promising results. Thus, many types of microalgae have turned out to be useful as part of the diet, increasing growth (due to protein deposition) and improving physiological activity, stress response, tolerance to starvation, resistance to diseases, and flesh quality [8]. In the present work, low inclusion levels of *N. gaditana* were used in diets containing a low percentage of fishmeal (15%), in order to assess their possible influence on gilthead seabream growth. At day 40 of the experiment, BL and BW values were higher in R5 group than in the other groups, while after 64 and 90 days, the highest values for BL and BW were recorded in R2.5 group, more appreciably for BW. When we did the analysis of fish morphology taking into account two space dimensions, the growth trajectory of control fish showed that neither PC1 nor PC2 increased through time. In fact, other studies in gilthead seabream reported a non-linear shape change trend during its ontogenesis [37]. Our data showed that the initial population of fish (control at day 0) had a higher PC1 and a lower PC2 values than control fish at days 40 and 64. However, control fish at day 40 showed higher PC1 and a lower PC2 values than at day 64. This data can be explained because gilthead seabream shows differential allometric growth rates of head, trunk, and tail depending on the age. Thus, at juveniles stages, the major shape changes that occurs is the widening of the trunk [38], characterized by a high PC2 value in our study. In fact, the increase in height within size is related to benthic feeding behavior, as well as to an increase in the development of the digestive organs, particularly the gut length. The growth of the gut length will allow the digestive of plant fragment that might be ingested in a more benthic feeding habitats [39,40]. The relation between the development of the digestive system and the increase in wide of the fish (high PC2 value) could explain why the fish that had been fed with the diets with higher proportion of microalgae (R5 and H5) always had a higher PC2 value than the H2.5 and R2.5 fish at all sampling points, being this difference highly significant between H5 and H2.5 after 90 days of feeding. Thus, we can conclude that the use of diets with microalgae for gilthead seabream might affect the development of the gut and, in turn, the shape of the specimens, triggering an earlier acquisition of a discoid body shape. Interestingly, a discoid body shape improves the swimming maneuverability and allows benthic feeding behavior as previously described in several fish species [38,41,42]. In fact, farmed gilthead seabream also showed a higher PC2 value than their wild counterparts of two different locations [28]. On the other hand, the fish that had been feed with diets with low inclusion of microalgae (R2.5 and H2.5) showed higher PC1 values than control fish at all sampling points as expected in growing fish. According to these results, we can conclude that the inclusion of *N. gaditana* in juvenile gilthead seabream diets accelerates the acquisition of a discoid body shape (decrease the isometric size and increase de allometric shape) in an inclusion level dependent-trend, but without affecting the total growth, since the conversion rates, growth rates, and daily intake were similar in all groups.

Other studies have also shown positive effects of microalgae on fish growth in a wide range of inclusion levels in diets. Thus, the use of *Arthrospira sp.* as a substitute for fishmeal in the range of 5 to 50% produced a favorable effect on fish growth [43,44,45,46,47]. Other microalgae species were able to replace fishmeal in the 6–20% range: for example, *Phaedactylum tricornutum* or a combination of *Nannochloropsis sp.* and *Isochrysis* sp. in Atlantic salmon [48], *Tetraselmis suecica* or *Isochrysis sp.* in European seabass [12,13], and *Scenedesmus almeriensis*, *Tetraselmis suecica*, and *Tisochrysis lutea* in gilthead seabream [14,15].

On the other hand, the present study also reveals that *N. gaditana* can be partially used as an alternative ingredient for replacing vegetable products (mainly soybean meal), which are usually used at high levels, as a substitute for fishmeal. Similarly, in juvenile gilthead seabreams, other authors studied the effect of adding 10% of *Tetraselmis*, as a partial replacement for soybean meal, in feeds with 20% fishmeal, for 61 days [49]. At the end of the experiment, the final weight, daily growth rate, conversion rate, protein efficiency index, body composition, and nutrient retention of the seabream did not differ owing to the diet [49]. Similarly, in our study, the conversion rates, growth rates, and daily intake were similar in all groups, and the survival values were high, being that these data were in agreement with previous results in this species during the pre-fattening and fattening onset phases [50].

Treatment of microalgae with cellulase enzymes to improve the availability of nutrients from *N. gaditana* did not have a significant effect on body growth parameters of gilthead seabream. However, when comparing the morphology of the fish that had been fed with raw microalgae with fish that had been fed with hydrolyzed microalgae at the same inclusion level, we observed significant differences in shape (PC2) between R2.5 and H2.5 groups after 40 days but not after 64 and 90 days. At this last point, no difference in isometric size (PC1) was observed between R2.5 and H2.5 fish. In contrast, differences in shape (PC2) were observed between R5 and H5 groups, at any sampling time of the experiment but not always with the same trend. However, from 64 days onwards, the fish that had been fed with the H5 diet showed a more discoid body shape (a statistically significant lower PC1 and a higher PC2) than the fish that had been fed with R5 diet. These data suggest that the inclusion of hydrolyzed microalgae in gilthead seabream diets at 5% level affects the geometric morphology of fish. As previously discussed, the relation between shape and gut development [38,40] suggests that the availability of nutrients from microalgae also affects the digestive system and probability also other systems, such as the immune or even the reproductive system. Further studies are being performed in our laboratory in order to clarify these relations.

### 4.2. Influence of the Diet on the Muscle Growth

The studies of the effect of the microalgae-supplemented diet on fish muscle growth are still very scarce. Recently, some authors studied the influence of *N. oceanica* on the muscle cellularity of spotted wolffish (*Anarhichas minor*) for 12 weeks, and they found no significant differences in growth of the white muscle or fast muscle cellularity among the feeding groups [1].

In the present work, the values of the white muscle transverse area at day 40 were similar in fish that had been fed with the different dietary treatments, with the exception of the H2.5 group, which showed the lowest values. However, after 90 days, the transverse area of the white muscle showed the following trend: C > R2.5 > R5 > H2.5 > H5. These groups showed differences in the fibrillar constitution of the myotome, with the greatest number of fibers (hyperplasia) in C, while hypertrophy was higher in R2.5. This fact shows the plasticity of the muscle cellularity of gilthead seabream with respect to the rearing factors (feeding regime, temperature, etc.), as usually observed in teleosts [29,30,51,52,53,54]. The variation in the fibrillar constitution of the myotome in the different groups might persist in more advanced stages of age [29,30,54], which can influence the texture of the fish fillet at commercial size, as seen in other species [30,55,56,57,58].

On the other hand, H5 and R5 groups had the highest proportion of small-middle size fibers, that could indicate a greater potential for muscle fiber formation in later stages of growth. Similarly, recent studies on microalgae replacement in fish diets [1,58] did not find significant changes on fibers size distribution but observed a trend to have smaller middle-size fibers on those fish fed with microalgae, suggesting that the potential for the formation of new fibers might be increased. While it is not clear how the inclusion of microalgae on the diets might increase the formation of new fibers, studies in humans have suggested that DHA and EPA can promote skeletal muscle growth by regulating the activation of the mTOR (mammalian Target of Rapamycin) pathway [59].

Long-term studies would be now necessary to assess the growth potential of the different feeding groups. For this reason, the feeding groups of this study are being maintained by our research team at the Centro Oceanográfico de Murcia (Mazarrón, Spain), in order to evaluate the possible effects of the experimental diets during pre-fattening and the onset of fattening at commercial size.

Regarding the enzymatic hydrolysis of microalgae, no significant effect on muscle parameters was observed in the present study.

## 5. Conclusions

1. The highest body values were reached in the R2.5 group, which seems to indicate that the lowest level of inclusion of raw *N. gaditana* (2.5%) is sufficient to obtain adequate growth of juvenile gilthead seabream.
2. The inclusion of *N. gaditana* in juvenile gilthead seabream diets accelerated the acquisition of a discoid body shape in an inclusion level-dependent trend, but without affecting the total growth, since the conversion rates, growth rates and daily intake were similar in all groups.
3. The inclusion of hydrolyzed microalgae in gilthead seabream diets at 5% level affected the geometric morphology of fish compared to raw microalgae at the same inclusion level.
4. The muscle plasticity of gilthead seabream gave rise to differences in the muscular constitution among the groups, with the highest hyperplasia values in C group, and the highest hypertrophy values in R2.5 group. The groups with higher levels of microalgae inclusion (R5 and H5) showed a higher percentage of small and medium-sized fibers, which may indicate a greater potential for growth in later stages of cultivation. Long-term studies are now necessary to check the effects on subsequent stages of growth.

## Figures and Tables

**Table 1:** Ingredient composition of the experimental diets.

Ingredient Composition (% Dry Matter)			Diets		
	C	R2.5	R5	H2.5	H5
Fish meal LT94 ^1^	15.0	15.0	15.0	15.0	15.0
Raw *N. gaditana*		2.5	5.0		
Hydrolyzed *N. gaditana*				2.5	5.0
Squid meal ^2^	2.0	2.0	2.0	2.0	2.0
CPSP90 ^3^	1.0	1.0	1.0	1.0	1.0
Krill meal ^4^	2.0	2.0	2.0	2.0	2.0
Gluten meal ^5^	15.0	15.0	15.0	15.0	15.0
Soybean protein concentrate ^6^	40.0	38.8	37.3	38.8	37.3
Fish oil ^7^	11.4	11.0	10.5	11.0	10.5
Soybean lecithin ^8^	1.0	1.0	1.0	1.0	1.0
Wheat meal ^9^	5.4	4.5	4.0	4.5	4.0
Choline chloride ^10^	0.5	0.5	0.5	0.5	0.5
Betain ^11^	0.5	0.5	0.5	0.5	0.5
Lysine ^12^	1.5	1.5	1.5	1.5	1.5
Methionine ^13^	0.6	0.6	0.6	0.6	0.6
Vitamin and mineral premix ^14^	2.0	2.0	2.0	2.0	2.0
Vitamin C ^15^	0.1	0.1	0.1	0.1	0.1
Guar gum ^16^	2.0	2.0	2.0	2.0	2.0
Proximal analysis (% dry matter)					
Crude protein	45.7	47.6	48.1	46.0	47.6
Crude lipid	15.0	15.7	15.3	15.9	15.9
Ash	7.4	7.6	8.0	7.7	7.4
Fiber	3.7	3.4	3.3	3.4	3.3
Nitrogen-free extract	28.2	25.7	25.3	27.0	25.8

^1^ 69.4% crude protein, 12.3% crude lipid (Norsildemel, Bergen, Norway); ^2, 3, 4^ Bacarel (UK); ^5^ 78% crude protein (Lorca Nutricion Animal SA, Murcia, Spain); ^6^ 65% crude protein, 8% crude lipid (DSM, France); ^7^ AF117DHA (Afamsa, Spain); ^8^ P700IP (Lecico, DE); ^9^ Local provider (Almería, Spain); ^10, 11,12, 13^ Lorca Nutricion Animal SA (Murcia, Spain); ^14^ Life bioencapsulation SL (Almería, Spain). Vitamins (mg kg^−1^): vitamin A (retinyl acetate), 2,000,000 international units (IU); vitamin D3 (DL-cholecalciferol), 200,000 IU; vitamin E (Lutavit E50), 10,000 mg; vitamin K3 (menadione sodium bisulphite), 2500 mg; vitamin B1(thiamine hydrochloride), 3000 mg; vitamin B2 (riboflavin), 3000 mg; calcium pantothenate, 10,000 mg; nicotinic acid, 20,000 mg; vitamin B6 (pyridoxine hydrochloride), 2000 mg; vitamin B9 (folic acid), 1500 mg; vitamin B12 (cyanocobalamin), 10 mg vitamin H (biotin), 300 mg; inositol, 50,000 mg; betaine (Betafin S1), 50,000 mg. Minerals (mg kg^−1^): Co (cobalt carbonate), 65 mg; Cu (cupric sulphate), 900 mg; Fe (iron sulphate), 600 mg; I (potassium iodide), 50 mg; Mn (manganese oxide), 960 mg; Se (sodium selenite), 1 mg; Zn (zinc sulphate) 750 mg; Ca (calcium carbonate), 18.6%; (186,000 mg); KCl, 2.41%; (24,100 mg); NaCl, 4.0% (40,000 mg); ^15^ TECNOVIT , Tarragona, Spain; ^16^ EPSA, Valencia, Spain. C, R2.5, R5, H2.5, H5 stand for the experimental diets, as explained in M&M Section.

**Table 2:** Fish body growth parameters at day 40 of the feeding trial.

Parameters	Experimental Groups				
	C	R2.5	R5	H2.5	H5
BL (cm)	11.76 ± 0.06 ^a^	11.90 ± 0.05 ^ab^	12.02 ± 0.06 ^b^	11.80 ± 0.04 ^a^	11.80 ± 0.02 ^a^
BW (g)	23.65 ± 0.35 ^ab^	23.60 ± 0.31 ^ab^	24.55 ± 0.31 ^b^	23.13 ± 0.27 ^a^	24.10 ± 0.23 ^ab^
CF (g cm^−3^)	1.46 ± 0.01 ^a^	1.43 ± 0.00 ^ab^	1.40 ± 0.01 ^b^	1.46 ± 0.00 ^a^	1.47 ± 0.01 ^a^
FCR	1.16 ± 0.02 ^a^	1.12 ± 0.08 ^a^	1.09 ± 0.02 ^a^	1.23 ± 0.10 ^a^	1.19 ± 0.03 ^a^
SGR (% d^−1^)	1.90 ± 0.02 ^a^	1.96 ± 0.10 ^a^	2.02 ± 0.02 ^a^	1.93 ± 0.05 ^a^	1.96 ± 0.12 ^a^

Parameters: body length (BL), body weight (BW), condition factor (CF), feed conversion rate (FCR), and specific growth rate (SGR), of all groups, at day 40 of the feeding trial. Different lower-case letters superscripts among groups within each row indicate significant differences (*p* < 0.05) for each parameter. Values are expressed as mean ± SEM.

**Table 3:** Fish body growth parameters at day 64 of the feeding trial.

	Experimental Groups				
Parameters	C	R2.5	R5	H2.5	H5
BL (cm)	12.87± 0.04 ^a^	13.09 ± 0.03 ^b^	13.06 ± 0.03 ^b^	12.90 ± 0.04 ^a^	12.92 ± 0.03 ^a^
BW (g)	33.56 ± 0.26 ^a^	34.77 ± 0.25 ^b^	33.13 ± 0.33 ^a^	33.40 ± 0.28 ^a^	33.84 ± 0.24 ^ab^
CF (g cm^−3^)	1.56 ± 0.02 ^ab^	1.55 ± 0.00 ^ab^	1.47 ± 0.01 ^a^	1.55 ± 0.03 ^ab^	1.58 ± 0.01 ^b^
FCR	1.26 ± 0.00 ^a^	1.17 ± 0.08 ^a^	1.43 ± 0.00 ^a^	1.28 ± 0.00 ^a^	1.26 ± 0.10 ^a^
SGR (% d^−1^)	1.45 ± 0.02 ^a^	1.54 ± 0.14 ^a^	1.30 ± 0.00 ^a^	1.42 ± 0.00 ^a^	1.47 ± 0.12 ^a^
DIR (% d^−1^)	1.33 ± 0.00 ^a^	1.29 ± 0.01 ^a^	1.38 ± 0.00 ^a^	1.49 ± 0.22 ^a^	1.37 ± 0.07 ^a^

Parameters: body length (BL), body weight (BW), condition factor (CF), feed conversion rate (FCR), specific growth rate (SGR), and daily intake rate (DIR) after 64 d of the feeding trial. Different lower-case letters superscripts among groups within each row indicate significant differences (*p* < 0.05) for each parameter. Values are expressed as mean ± SEM.

**Table 4:** Fish body growth parameters at day 90 of the feeding trial.

	Experimental Groups				
Parameters	C	R2.5	R5	H2.5	H5
BL (cm)	14.40 ±0.10	14.63 ± 0.05	14.43 ± 0.10	14.45 ± 0.05	14.5 ± 0.05
BW (g)	49.10 ± 0.60	51.30 ± 0.50	49.50 ± 0.60	49.90 ± 0.60	50.2 ± 0.60
CF (g cm^−3^)	1.64 ± 0.01	1.64 ± 0.02	1.65 ± 0.01	1.65 ± 0.02	1.65 ± 0.03
FCR	1.07 ± 0.00	1.04 ± 0.01	1.01 ± 0.02	1.01 ± 0.01	1.01 ± 0.02
SGR (% d^−1^)	1.47 ± 0.01	1.49 ± 0.00	1.54 ± 0.00	1.54 ± 0.01	1.52 ± 0.01
DIR (% d^−1^)	1.31 ± 0.02	1.29 ± 0.03	1.28 ± 0.01	1.29 ± 0.01	1.28 ± 0.01

Parameters: body length (BL), body weight (BW), condition factor (CF), feed conversion rate (FCR), specific growth rate (SGR), and daily intake rate (DIR) after 90 d of the feeding trial. These parameters did not show significant differences among the experimental groups at day 90. Values are expressed as mean ± SEM.

**Table 5:** Morphological traits of gilthead seabream at day 0, and after feeding the experimental diets over 40 days.

Measurements	Day 0	Day 40				
	C	C	R2.5	R5	H2.5	H5
SL	8.03 ± 0.08	9.70 ± 0.08	9.89 ± 0.08	9.88 ± 0.08	9.71 ± 0.06	9.84 ± 0.06
TPM-MCH	1.28 ± 0.02	1.43 ± 0.02	1.45 ± 0.02	1.49 ± 0.02	1.51 ± 0.02	1.49 ± 0.02
MCH-ADF	2.49 ± 0.04	1.99 ± 0.09	2.04 ± 0.04	2.01 ± 0.07	2.67 ± 0.05	2.76 ± 0.04
ADF-PDF	4.22 ± 0.04	6.02 ± 0.09	6.14 ± 0.06	6.16 ± 0.09	5.33 ± 0.05	5.41 ± 0.05
PDF-PAF	0.36 ± 0.12	1.00 ± 0.02	0.98 ± 0.01	0.97 ± 0.01	0.97 ± 0.01	1.00 ± 0.01
PAF-AAF	1.75 ± 0.03	2.29 ± 0.05	2.40 ± 0.03	2.43 ± 0.03	2.43 ± 0.04	2.51 ± 0.03
AAF-PF	2.45 ± 0.03	2.77 ± 0.05	2.78 ± 0.04	2.80 ± 0.03	2.61 ± 0.04	2.72 ± 0.03
PF-OP	1.56 ± 0.02	2.26 ± 0.0	2.25 ± 0.04	2.18 ± 0.04	2.12 ± 0.04	2.12 ± 0.03
OP-TPM	1.74 ± 0.04	1.95 ± 0.03	1.96 ± 0.04	1.98 ± 0.04	2.06 ± 0.03	2.04 ± 0.03
OP-DPF	1.63 ± 0.04	2.22 ± 0.05	2.24 ± 0.03	2.19 ± 0.03	2.14 ± 0.05	2.18 ± 0.03
ED	˂1 ± 0.00	1.02 ± 0.01	1.00 ± 0.01	1.01 ± 0.01	1.02 ± 0.03	1.00 ± 0.01
MHC-OP	1.99 ± 0.03	2.33 ± 0.03	2.35 ± 0.03	2.38 ± 0.03	2.41 ± 0.03	2.41 ± 0.02
MHC-PF	3.12 ± 0.05	4.00 ± 0.04	4.01 ± 0.03	3.96 ± 0.03	3.94 ± 0.03	3.98 ± 0.02
MHC-AAF	5.06 ± 0.05	6.22 ± 0.06	6.24 ± 0.05	6.20 ± 0.05	6.00 ± 0.05	6.10 ± 0.04
ADF-PF	3.08 ± 0.04	3.76 ± 0.04	3.75 ± 0.03	3.76 ± 0.03	3.71 ± 0.02	3.81 ± 0.03
ADF-AAF	3.71 ± 0.04	5.16 ± 0.08	5.14 ± 0.05	5.13 ± 0.06	4.60 ± 0.04	4.68 ± 0.04
ADF-PAF	4.57 ± 0.04	6.44 ± 0.08	6.52 ± 0.06	6.52 ± 0.08	5.78 ± 0.05	5.88 ± 0.05

Measurement abbreviations explained in Figure 1.

**Table 6:** Morphological traits of gilthead seabream fed the experimental diets over 64 days.

	64 Days				
Measurements	C	R2.5	R5	H2.5	H5
SL	11.19 ± 0.06	11.36 ± 0.05	11.15 ± 0.07	11.03 ± 0.06	11.05 ± 0.06
TPM-MCH	1.90 ± 0.03	1.75 ± 0.02	1.63 ± 0.03	1.77 ± 0.02	1.56 ± 0.02
MCH-ADF	2.97 ± 0.06	3.01 ± 0.06	3.36 ± 0.06	3.07 ± 0.05	2.85 ± 0.05
ADF-PDF	6.03 ± 0.06	6.19 ± 0.06	5.86 ± 0.08	5.94 ± 0.05	6.39 ± 0.05
PDF-PAF	1.21 ± 0.02	1.24 ± 0.02	1.20 ± 0.02	1.19 ± 0.01	1.18 ± 0.01
PAF-AAF	2.78 ± 0.04	2.81 ± 0.03	2.66 ± 0.04	2.50 ± 0.04	2.42 ± 0.03
AAF-PF	3.41 ± 0.05	3.45 ± 0.04	3.51 ± 0.05	3.49 ± 0.04	3.45 ± 0.04
PF-OP	2.37 ± 0.03	2.33 ± 0.03	2.22 ± 0.03	2.32 ± 0.03	2.35 ± 0.03
OP-TPM	2.20 ± 0.03	2.27 ± 0.03	2.20 ± 0.03	2.27 ± 0.02	2.25 ± 0.02
OP-DPF	2.57 ± 0.03	2.60 ± 0.02	2.40 ± 0.03	2.47 ± 0.02	2.32 ± 0.02
ED	1.04 ± 0.01	1.05 ± 0.01	1.02 ± 0.01	1.06 ± 0.01	1.06 ± 0.01
MHC-OP	2.74 ± 0.02	2.74 ± 0.03	2.65 ± 0.02	2.75 ± 0.02	2.66 ± 0.02
MHC-PF	4.29 ± 0.03	4.37 ± 0.03	4.27 ± 0.03	4.37 ± 0.03	4.44 ± 0.03
MHC-AAF	6.90 ± 0.05	7.08 ± 0.04	7.10 ± 0.05	7.10 ± 0.05	7.26 ± 0.05
ADF-PF	4.37 ± 0.03	4.36 ± 0.03	4.28 ± 0.03	4.35 ± 0.03	4.27 ± 0.03
ADF-AAF	5.39 ± 0.05	5.52 ± 0.04	5.33 ± 0.05	5.47 ± 0.05	5.74 ± 0.04
ADF-PAF	6.65 ± 0.06	6.84 ± 0.06	6.50 ± 0.06	6.56 ± 0.06	6.89 ± 0.05

Measurement abbreviations explained in Figure 1.

**Table 7:** Morphological traits of gilthead seabream fed the experimental diets over 90 days.

	Experimental Diets				
Measurements	C	R2.5	R5	H2.5	H5
SL	12.01 ± 0.07	12.03 ± 0.07	11.99 ± 0.08	12.20 ± 0.07	12.12 ± 0.06
TPM-MCH	1.61 ± 0.02	1.56 ± 0.02	1.54 ± 0.02	1.64 ± 0.02	1.59 ± 0.02
MCH-ADF	3.64 ± 0.04	3.86 ± 0.04	3.75 ± 0.04	3.60 ± 0.04	3.56 ± 0.05
ADF-PDF	5.89 ± 0.05	5.73 ± 0.05	5.84 ± 0.06	6.11 ± 0.06	6.20 ± 0.06
PDF-PAF	1.69 ± 0.02	1.69 ± 0.02	1.66 ± 0.02	1.71 ± 0.02	1.62 ± 0.02
PAF-AAF	2.36 ± 0.03	2.33 ± 0.03	2.31 ± 0.03	2.41 ± 0.04	2.46 ± 0.03
AAF-PF	3.90 ± 0.04	3.99 ± 0.04	3.92 ± 0.05	4.04 ± 0.05	3.94 ± 0.05
PF-OP	2.25 ± 0.03	2.25 ± 0.03	2.23 ± 0.03	2.17 ± 0.04	2.25 ± 0.03
OP-TPM	2.25 ± 0.03	2.20 ± 0.03	2.21 ± 0.03	2.33 ± 0.02	2.20 ± 0.03
OP-DPF	2.28 ± 0.04	2.20 ± 0.03	2.30 ± 0.04	2.35 ± 0.03	2.34 ± 0.03
ED	0.98 ± 0.01	0.97 ± 0.01	0.99 ± 0.01	0.98 ± 0.01	0.98 ± 0.01
MHC-OP	2.85 ± 0.02	2.81 ± 0.03	2.79 ± 0.03	2.96 ± 0.03	2.83 ± 0.03
MHC-PF	4.43 ± 0.03	4.42 ± 0.03	4.42 ± 0.04	4.53 ± 0.04	4.45 ± 0.03
MHC-AAF	7.52 ± 0.05	7.53 ± 0.05	7.52 ± 0.06	7.75 ± 0.05	7.54 ± 0.05
ADF-PF	4.77 ± 0.04	4.82 ± 0.03	4.78 ± 0.04	4.88 ± 0.03	4.75 ± 0.04
ADF-AAF	5.71 ± 0.05	5.64 ± 0.04	5.66 ± 0.07	5.96 ± 0.05	5.82 ± 0.04
ADF-PAF	6.62 ± 0.05	6.46 ± 0.04	6.52 ± 0.07	6.87 ± 0.05	6.78 ± 0.05

Measurement abbreviations explained in Figure 1.

**Table 8:** Component loadings, percent of variance (% V), Eigen values and two-way ANOVA significance for the principal component analysis (with varimax rotation).

Measurements		PC1	PC2
SL		0.721	0.690
TPM-MCH		0.722	0.684
MCH-ADF		0.742	0.664
ADF-PDF		0.708	0.701
PDF-PAF		0.720	0.688
PAF-AAF		0.702	0.703
AAF-PF		0.746	0.661
PF-OP		0.661	0.747
OP-TPM		0.743	0.664
OP-DPF		0.665	0.741
ED		0.712	0.694
MCH-OP		0.740	0.668
MCH-PF		0.713	0.698
MCH-AAF		0.730	0.680
ADF-PF		0.729	0.687
ADF-AAF		0.723	0.687
ADF-PAF		0.719	0.692
Variance %		51.523	47.783
Cumulative %		51.523	99.306
Eigen values		16.864	0.018
ANOVA	Treatment	0.0000	0.0000
	Time	0.0000	0.0000
	Treatment × Time	0.0000	0.0000

Measurement abbreviations explained in Figure 1.

**Table 9:** Mean values of the transverse section of the white muscle of the different experimental groups at 0, 40, and 90 d of the feeding trial.

Section		Experimental Groups				
		C	R2.5	R5	H2.5	H5
B (mm^2^)	Day 0	122.7 ± 4.1				
	Day 40	212.0 ± 8.3 ^a^	206.48 ± 8.1 ^ab^	210.5 ± 6.3 ^a^	177.2 ± 5.9 ^b^	206.0 ± 9.4 ^ab^
	Day 90	391.2 ±13.6 ^a^	358.9 ± 14.7 ^ab^	341.2 ± 13.0 ^ab^	340.1 ± 12.9 ^ab^	332.8 ± 9.9 ^b^

B: transverse area of the white muscle. Different lower-case letters superscripts in each row indicate significant differences (*p* < 0.05) among the experimental groups, within each sampling time. Values are expressed as mean ± SEM.

**Table 10:** Mean values of the muscle cellularity of all groups at day 0 (beginning of the experiment) and 90 (end of the feeding trial).

Groups	Day 0	Day 90				
	C	C	R2.5	R5	H2.5	H5
A (μm^2^)	1791.60 ± 1.94	2644.80 ± 524.30	3133.90 ± 167.60	2924.60 ± 78.03	2921.30 ± 112.90	2853.56 ± 67.41
D (μm)	35.93 ± 1.44	49.50 ± 1.60	48.80 ± 1.50	48.70 ± 0.80	47.56 ± 1.16	48.18 ± 0.84
N (×10^3^)	70.49 ± 4.32	120.87 ± 8.08	118.15 ± 9.63	117.03 ± 4.17	117.51 ± 7.35	116.16 ± 4.80
Dens	569.12 ± 31.04	309.20 ± 13.50	329.20 ± 21.70	344.04 ±8.80	346.87 ± 14.90	350.71 ± 9.19

At day 0 (beginning of the experiment), C represents the mean of all fish before being distributed in the experimental groups, while, on day 90, C represent the fish fed with the control diet (without microalgae). A: area of white fibers; D: Minimum diameter of white muscle fibers; N: number of white muscle fibers; Dens: fibrillar density (number of muscle fibers mm^−2^). These parameters did not show significant differences among the experimental groups. Values are expressed as mean ± SEM.

## Supplementary Materials

The following are available online at https://www.mdpi.com/2076-2615/10/12/2270/s1, Figure S1: Two-way ANOVA of the morphometric measurements.
