# Supplementary material for: Influence of Low Dietary Inclusion of the Microalga Nannochloropsis gaditana (Lubián 1982) on Performance, Fish Morphology, and Muscle Growth in Juvenile Gilthead Seabream (Sparus aurata)
Source: Animals (Basel). 2020 Dec 1;10(12):2270. doi: 10.3390/ani10122270 (PMC7761204; doi:10.3390/ani10122270)
Supplement: Supplementary file 1 [file animals-10-02270-s001.pdf]

Fig. S1 (supplementary data): two-way ANOVA of the morphometric measurements.

[1]Statistical limit established in P<0.05. Statistical significances marked in bold.

**Measurement**	**Comparison type**	**F value**	**Significance[1]**
SL	Treatment	8.920	0.558
	Time	1477.913	**0.000**
	Treatment*Time	3.362	**0.018**
TPM-MCH	Treatment	20.890	**0.000**
	Time	355.305	**0.000**
	Treatment*Time	34.611	**0.000**
MCH-ADF	Treatment	32.180	**0.000**
	Time	393.796	**0.000**
	Treatment*Time	77.294	**0.000**
ADF-PDF	Treatment	38.159	**0.000**
	Time	299.923	**0.000**
	Treatment*Time	63.812	**0.000**
PDF-PAF	Treatment	1.059	**0.003**
	Time	523.070	**0.000**
	Treatment*Time	1.394	**0.000**
PAF-AAF	Treatment	11.943	**0.006**
	Time	266.578	**0.000**
	Treatment*Time	43.167	**0.000**
AAF-PF	Treatment	2.833	**0.001**
	Time	795.336	**0.000**
	Treatment*Time	4.289	**0.073**
PF-OP	Treatment	6.653	**0.023**
	Time	205.781	**0.000**
	Treatment*Time	6.334	0.107
OP-TPM	Treatment	5.084	**0.000**
	Time	203.462	**0.000**
	Treatment*Time	1.799	**0.000**
OP-DPF	Treatment	16.989	**0.020**
	Time	347.792	**0.000**
	Treatment*Time	8.926	**0.000**
ED	Treatment	2.894	0.330
	Time	2169.556	**0.000**
	Treatment*Time	2.008	0.519
MHC-OP	Treatment	3.421	**0.000**
	Time	646.802	**0.000**
	Treatment*Time	7.881	**0.000**
MHC-PF	Treatment	5.498	0.111
	Time	778.222	**0.000**
	Treatment*Time	6.206	**0.031**
MHC-AAF	Treatment	4.955	**0.035**
	Time	1120.468	**0.000**
	Treatment*Time	12.757	**0.002**
ADF-PF	Treatment	1.852	**0.007**
	Time	1345.609	**0.000**
	Treatment*Time	6.422	0.122
ADF-AAF	Treatment	20.758	**0.000**

	Time	664.408	**0.000**
	Treatment*Time	63.790	**0.000**
ADF-PAF	Treatment	40.100	**0.000**
	Time	496.351	**0.000**
	Treatment*Time	47.629	**0.000**
